# Association between *IL1B* rs16944 polymorphism and the risk of idiopathic inflammatory myopathies

**DOI:** 10.3389/fimmu.2026.1697044

**Published:** 2026-04-01

**Authors:** Ignacio García-De la Torre, Irene Mendoza-Lujambio, Lilia Andrade-Ortega, Mónica Vazquez-Del Mercado, Karina Lamadrid-Gámez, Celso Pérez-Rostro, Daniel Pérez-Covarrubias

**Affiliations:** 1 Instituto de Investigación en Inmunología, Centro Universitario de Ciencias de la Salud, Universidad de Guadalajara, Guadalajara, Jalisco, Mexico; 2 Immunology and Rheumatology Department, Hospital General de Occidente, Secretaria de Salud Jalisco, Zapopan, Jalisco, Mexico; 3 Lab. De Genética humana. Sección de Estudios de Posgrado e Investigación, Escuela Superior de Medicina. Instituto Politécnico Nacional, Mexico City, Mexico; 4 Servicio de Reumatología, Centro Médico Nacional 20 de Noviembre, Instituto de Seguridad y Servicios Sociales de los Trabajadores del Estado (ISSSTE), Mexico City, Mexico; 5 División de Medicina Interna, Especialidad en Reumatología 004086, Nuevo Hospital Civil de Guadalajara Dr. Juan I. Menchaca, Guadalajara, Jalisco, Mexico; 6 Departamento de Biología Molecular y Genómica, Instituto de Investigación en Reumatología y del Sistema Músculo Esquelético, Centro Universitario de Ciencias de la Salud, Universidad de Guadalajara, Guadalajara, Jalisco, Mexico; 7 Escuela Superior de Medicina, Instituto Politécnico Nacional, Mexico City, Mexico; 8 Immunology Department, Centro de Estudios de Investigacion Basica y Clinica, Guadalajara, Jalisco, Mexico

**Keywords:** association, idiopathic, idiopathic inflammatory myopathies, *IL1B*, polymorphism, rs16944

## Abstract

**Introduction:**

Idiopathic inflammatory myopathies (IIMs) are autoimmune, systemic diseases that affect skeletal muscles. The causes are not fully understood. IIMs present with proximal weakness, elevated muscle enzymes (primarily creatine phosphokinase, or CPK), and multiorgan involvement, often pulmonary. Muscle tissue shows leukocyte infiltration, and specific autoantibodies are present. We aimed to investigate the possible association between the *IL1B* rs16944 polymorphism and the risk of developing IIMs.

**Methods:**

DNA was extracted from blood samples collected from 57 patients with IIMs and 50 healthy controls. The *IL1B* rs16944 polymorphism was analyzed. Myositis-specific autoantibodies (MSAs), myositis-associated autoantibodies (MAAs), and antinuclear antibodies (ANAs) were determined.

**Results:**

The IIM subtypes in our patients were dermatomyositis (DM), polymyositis, amyopathic DM, juvenile DM, cancer-associated myositis, scleromyositis, and antisynthetase syndrome. The three most frequent were DM (47.4%), polymyositis (22.8%), and cancer-associated myositis (17.5%). Regarding ANAs, the three most frequent patterns in patients were AC-4 (nuclear fine speckled, 40%), AC-21 (cytoplasmic reticular/AMA, 10%), and AC-19 (cytoplasmic dense fine speckled, 8.6%), while 15.7% were negative. About myositis autoantibodies, the most frequent MSAa in IIMs were anti-SAE1 (12.5%), anti-MDA5 (10.7), and anti-TIF1g (10.7%). The most common MAAs were as follows: anti-Ro52 (30.4%), anti-Ku (14.3%), and anti-PM100 (5.4%). Analysis of the *IL1B* polymorphism rs16944 revealed a significant association with susceptibility to IIMS in the codominant model (CC vs. CT, OR = 4.6136, 95% CI: 1.83–11.65, *p* = 0.0012), dominant model (OR = 3.432, 95% CI: 1.47–7.98, *p* = 0.0044) and overdominant model (OR = 3.587, 95% CI: 1.58–7.92, *p* = 0.0021). Considering the female group, between cases and HC, the codominant (CC vs. CT), the dominant, and the overdominant genetic models presented significant differences (*p* = 0.0015, 0.0038, and OR = 4.5043, 95% CI: 1.59–12.68, *p* = 0.0044, respectively). Also, a significant association could be observed with DM, polymyositis (PM), DM+PM, juvenile DM, amyopathic DM, antisynthetase syndrome (ASyS), and cancer-associated myositis (CAM). Enzyme levels of CPK presented significant differences in our IIMs patients. The rs16944 polymorphism is significantly associated with various clinicopathological characteristics.

**Conclusion:**

This study has, for the first time, revealed an association between the *IL1B* rs16944 CC (codominant and dominant models) and CCTT (overdominant) genotypes and the IIMs phenotype (OR = 4.6136, 3.4232, and 3.5387, respectively).

## Introduction

IIMs are a group of autoimmune and systemic diseases that affect skeletal muscles. These diseases are characterized by proximal weakness, elevated enzymes, particularly creatine phosphokinase (CPK), multiorgan involvement, usually pulmonary, leukocyte infiltrates in muscle tissue, and the presence of specific autoantibodies. These clinical, histological, and immunopathological characteristics allow them to be grouped into different types of dermatomyositis (DM), amyopathic DM, juvenile DM, polymyositis, inclusion body myositis, immune-mediated necrotizing myopathy, and juvenile myositis. IIMs generally occur in adults, except for juvenile DM (1–4).

The cause of IIMs is not yet fully understood. However, as with other autoimmune conditions, IIMs arise from chronic immune activation in genetically susceptible individuals following certain environmental stimuli (5). However, it has been observed that T cells and macrophages predominate in inflammatory infiltrates in muscle tissue from patients with these disorders. This finding in muscle, along with the presence of both myositis-specific antibodies (MSAs) and myositis-associated antibodies (MAAs), suggests that an autoimmune component is involved in the development of IIMs (6). The presence of MSAs has substantial prognostic value in these diseases, as they can be detected before the first clinical manifestations (7). Immunogenetic features, such as the common finding of autoantibodies that are related to disease activity, the immunopathology in altered tissues, and the therapeutic outcome of immune-modulating factors, reinforce an immune-mediated cause in IIMs (8–11).

IIMs are considered complex diseases with genetic components (12, 13). Numerous polymorphic genes involved in immune response have been associated with the IIMs, the most important being those that encode antigen-presenting proteins like human major histocompatibility complex (MHC, *HLA-A, -B, -Cw, -DR, -DQ, -DP*) and some other genes that play relevant roles in immune activation, such as *TNFA*, *IL1A*, *IL1B*, and *IL-1RN*, *IgG*, and *IgK* (14, 15).

**Figure 1:**
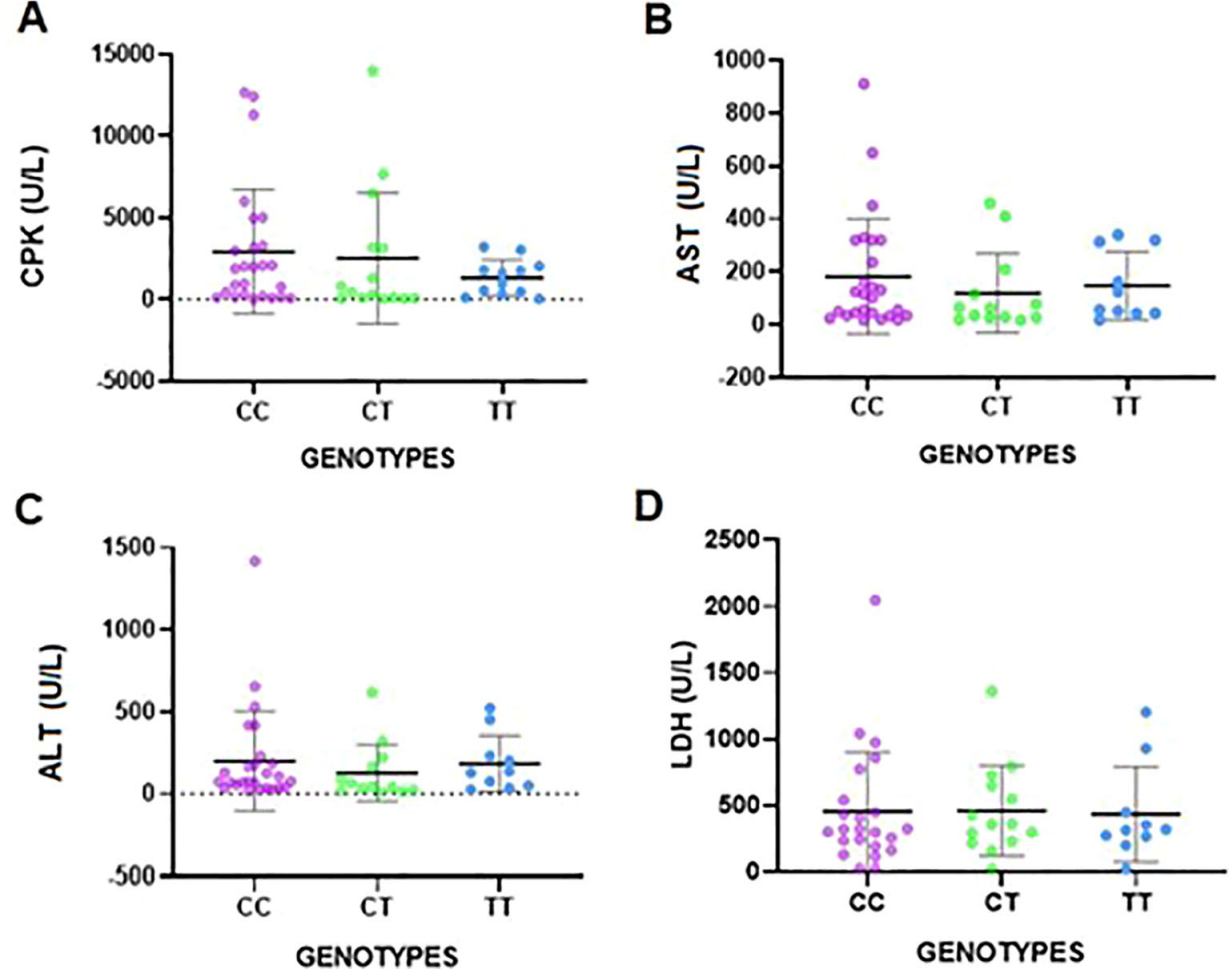
Enzyme levels of CPK, AST, ALT, and LDH analyzed by genotype. The bars represent the mean and standard deviation. A Student´s t-test was utilized to compare levels among groups. **(A)**. CPK (U/L). **(B)**. AST (U/L). **(C)**. ALT (U/L). **(D)**. LDH (U/L).

Interleukin-1B (IL-1B) is a member of the interleukin 1 (IL-1) family. The *IL1B* gene encodes interleukin-1β, a potent proinflammatory cytokine involved in various cellular functions (**Figure 1**), including cell proliferation, differentiation, and apoptosis (16). It plays a key role in rheumatic diseases with autoimmune involvement (17, 18), as it contributes to chronic inflammation and damage in several autoimmune conditions (19). *IL1B* polymorphisms influence IL-1β levels, and excess IL-1β is associated with the pathogenesis of autoimmune diseases, such as SLE and rheumatoid arthritis (20, 21). The *IL1B* rs16944 polymorphism is in the promoter region at nucleotide -511, and it is related to the secretion of the IL-1b protein (22). High expression of *IL-1B* is associated with the pathogenesis of autoimmune diseases like SLE and RA (23).

Polymorphisms in *IL1B* are associated with a higher risk of autoimmune diseases. However, the rs16944 polymorphism has not been studied in IIMs; therefore, it was selected for analysis of its role in IIMs susceptibility in the Mexican population.

## Methods

### Subjects

Fifty-seven patients with an IIMs diagnosis were included in the study. Subjects were recruited from Guadalajara, Jalisco, and Mexico City. The Institutional Committees for Research and Ethics in Research of the Hospital de Occidente (Zapopan, Jalisco) approved this study. All subjects were evaluated, met the Bohan and Peter criteria, and provided written informed consent (1, 24). All patients were Mexican mestizos with no biological relation to each other or to patients or controls. Myositis autoantibodies were measured by the Autoimmune Inflammatory Myopathies 16 Ag immunoblot EUROLINE panel (EUROIMMUN AG, Lübeck, Germany). Baseline laboratory studies included serum creatinine, phosphokinase (CPK), aspartate aminotransferase (AST), alanine aminotransferase (ALT), and lactate dehydrogenase (LDH). Clinical histories included heliotrope rash, V sign, shawl sign, Gottron papules, Holster sign, basal poikiloderma, basal panniculitis, basal perionyxis, Raynaud’s phenomenon, basal arthritis, basal hyperpigmentation, periungueal erythema, basal calcinosis, dysphagia, dyspnea, basal arrhythmia, vasculitis, mechanic’s hands, proximal weakness, distal weakness, interstitial lung disease, atrophy, necrosis, abnormal electromyography, inflammation, fat infiltration, endomysial infiltration, perimysial infiltration, and perivascular infiltration.

### Control group

Blood samples were collected from 50 healthy controls. These individuals were clinically healthy men and women, born as Mexican mestizos, unrelated to patients or other controls.

### DNA extraction

Whole blood was collected in EDTA, and DNA was extracted using a commercially available kit (Wizard^®^ Genomic DNA Purification Kit, Promega, USA) according to the manufacturer’s instructions. The DNA was kept at −20 °C until polymerase chain reaction (PCR) analysis.

### Genotyping

Genotypes were analyzed using a commercial TaqMan assay (Applied Biosystems, San Francisco, CA, USA) to evaluate the rs16944 polymorphism in the *IL1B* gene. The rs16944 genotypes are CC (dominant), CT (heterozygote), and TT (recessive). The genetic models analyzed included dominant, recessive, codominant, and overdominant. In the dominant model, CC is compared with CT+TT; in the recessive model, CC+CT with TT; in the codominant model, CC with CT, CT with TT, and CC with TT. The codominant model is the most general and assumes each genotype imparts a different, non-additive disease risk. The overdominant model compares CT with CC+TT, assuming the heterozygote has the greatest effect in a condition or disease.

### Statistical analysis

Differences between groups were assessed by comparing allele and genotype frequencies. These group differences in frequencies were analyzed using the chi-square test. Odds ratios (ORs) with 95% confidence intervals (CIs) were estimated to assess the strength of the association. For genotype groups with fewer than five individuals, Fisher’s exact test was used. The healthy control group met the Hardy-Weinberg equilibrium. A student’s test compared muscular enzyme levels among the three genotypes in IIM patients. Results were considered significant when *p* > 0.05. Analyses and graphics were performed using GraphPad^®^ Prism version 8.

## Results

### Demographic and clinical characteristics in the cases group

Fifty-seven patients with IIMs and 50 healthy subjects in the control group were included. The case group was predominantly composed of women with a 3.4:1 proportion (44 vs. 13, *p* = 0.0072). The IIMs found in the patients were DM, polymyositis (PM), amyopathic dermatomyositis (ADM), juvenile dermatomyositis (JDM), scleromyositis (SM), antisynthetase syndrome (ASyS), cancer-associated myositis (CAM), overlap myositis (OM), and Immune-mediated necrotizing myositis (IMNM). The three most frequent IIMs found in our cohort are dermatomyositis (27, 47.4%), polymyositis (13, 22.8%), and cancer-associated myositis (10, 17.5%). The frequency of DM+PM is 70.2% (*n* = 40). When the types of IIMs were compared between genders, significant differences were found in DM+PM, DM, and PM (*p* = 0.0016, 0.0140, and 0.0099, respectively). The mean age was 54.6 (**Table 1**).

**Table 1:** Baseline characteristics of the IIM group.

DEMOGRAPHICS	TOTAL n	FEMALE n	MALE n	OR	95% CI	p
Healthy controls, n (%)	50	26 (52)	24 (48)			
Age, media (SD)	47.9 (18.8)	47.8 (20.9)	48.1 (16.5)			
Rate F:M	1.1:1					
Patients, n (%)	57	44 (77.2)	13 (22.8)	**3.1243**	**1.36-7.17**	**0.0072^a^**
Age, media (SD)	54.6 (16.5)	55.6 (15.7)	51.4 (19.2)			
Rate F:M	3.4:1					
Tobacco consumption	7	3	4			**0.0407^c^**
Alcohol consumption	5	0	5			**0.0003^c^**
Hypertension	15	12	3			NS
T2D	10	7	3			NS
CPK U/L (SD)	2454.2 (3444.2)	1795.2 (2438.4)	4346.1 (5168.6)			**0.0179^b^**
AST U/L (SD)	158.5 (184.8)	127.3 (147.2)	237.1 (251.8)			NS^b^
ALT U/L (SD)	180.4 (246.5)	134.1 (162.8)	300.8 (378.2)			**0.0332^b^**
LDH U/L (SD)	452.3 (391.8)	406.6 (330.8)	582.3 (518.7)			NS^b^
DIAGNOSTIC						
Healthy controls	50	26	24			
IIMs	57	44	13	**3.1243**	**1.36-7.17**	**0.0072^a^**
DM+PM n	40	34	6	**5.2308**	**1.87-14.65**	**0.0016^a^**
DM n	27	22	5	**4.0615**	**1.33-12.43**	**0.0140^a^**
PM n	13	12	1			0.0099^c^
ADM n	3	3	0			NS^c^
JDM n	7	5	2			NS^c^
SM n	3	0	3			NS^c^
ASyS n	3	1	2			NS^c^
CAM n	10	8	2			NS^c^
OM n	5	3	2			NS^c^
IMNM n	1	1	0			NS^c^

CPK, Creatine phosphokinase; AST, Aspartate aminotransferase; ALT, Alanine aminotransferase; LDH, Lactic dehydrogenase; U/L, Units per Liter; IIMs, Idiopathic inflammatory myopathies; DM, Dermatomyositis; PM, Polymyositis; ADM, Amyopathic dermatomyositis; JDM, Juvenile dermatomyositis; SM, Scleromyositis; ASyS, Antisynthetase syndrome; CAM, Cancer-associated myositis; OM, Overlap myositis; IMNM, Immune-mediated necrotizing myopathy. a) OR (Chi-square). b) One way ANOVA. c) Fisher’s exact test. Bold values indicate a significant difference (*p* >0.05).

The comparison by gender distribution of the demographic and clinical variables, such as type 2 diabetes, hypertension, smoking, and drinking habits, showed significant differences in tobacco and alcohol consumption (*p* = 0.0407 and 0.0003, respectively). This finding was also observed in serum enzyme levels (CPK, creatine phosphokinase; AST, aspartate aminotransferase; ALT, alanine aminotransferase; LDH, lactic dehydrogenase), where CPK and ALT showed higher levels in males (p = 0.0179 and 0.0332, respectively) (**Table 1**).

In regard to the antinuclear antibodies (ANAs), eight patterns were found, and the three most frequent in IIMs patients were AC-4, nuclear fine speckled (40%); AC-21 cytoplasmic reticular/AMA (10%); and AC-19, cytoplasmic dense fine speckled (8.6%), while 10% presented more than 1 pattern (mixed) and 15.7% were negative. AC-4 was the only pattern that showed a significant difference between genders, being more frequent in female (*p* = 0.0047) (**Supplementary Table 1**). When the genotypic comparison was done, the AC-4 pattern presented significant differences in the codominant model (CC vs CT), (*p* = 0.0155). Also, this significant difference was observed in the mixed ANAs pattern, and with the positives for ANAs, in the codominant model (CC vs CT) (*p* = 0.0298, and *p*= 0.0459, respectively) (**Supplementary Table 1**).

When myositis autoantibodies were analyzed, the most frequent MSAs in IIMs were anti-SAE1 (12.5%), anti-MDA5 (10.7) and anti-TIF1g (10.7%). The most common MAAs were: anti-Ro52 (30.4%), anti-Ku (14.3%), and anti-PM100 (5.4%). When a comparison between genders was performed, non-significant differences were obtained. However, when the genotype frequencies were analyzed, the dominant genetic model showed significant differences in all MSAs and in the autoantibody anti-OJ (*p* = 0.0256 and *p* = 0.0428, respectively) (**Supplementary Table 2**). When the enzyme levels of CPK, AST, ALT and LDH were analysed by genotype, only in CPK the differences among genotypes where significant (*p* = 0.0003) (**Figure 1** and **Table 2**).

**Table 2:** Genotypic analysis in demographic and enzyme levels in patients with IIMs.

FEATURE	TOTAL	CC	CT	TT	MODEL	*p*
TOTAL	57	28	16	13		
Female, n (%)	44 (77.2)	21 (75)	12 (75)	11 (84.6)		
Male, n (%)	13 (22.8)	7 (25)	4 (25)	2 (15.4)	ALL COD MODELS	NS^a^
Age, n (SD)	54.6 (16.5)	61.7 (8.4)	43.7 (22.4)	52.5 (14.8)		0.0017^b^
					CC vs CT	0.0012^b^
					CC vs TT	**0.0315^b^**
					CT vs TT	NS^b^
CPK (U/L)	2409.3 (3427.52)	2825.1 (3754.2)	2527.6 (4000.6)	1325.8 (1093.2)		**0.0003^a^**
AST (U/L)	155.9 (183.8)	176.7 (215.2)	119.3 (150.1)	147.1 (129.9)	ALL COD MODELS	NS^b^
ALT (U/L)	177.4 (244.9)	196.8 (294.6)	129.5 (176.0)	187.4 (173.0)	ALL COD MODELS	NS^b^
LDH (U/L)	450.6 (387.8)	452.2 (437.7)	460.0 (340.4)	433.4 (357.5)	ALL COD MODELS	NS^b^

NS, Non significant; COD, Codominant model; DOM, Dominant model; OD, Overdominant model; CPK, Creatine phosphokinase; AST, Aspartate aminotransferase; ALT, Alanine aminotransferase; LDH, Lactic dehydrogenase. a) Fisher’s exact test. b) One-way ANOVA. Bold values indicate a significant difference (*p* >0.05).

### Allelic and genotyping frequencies of the rs16944 polymorphism

The *IL1B* gene single-nucleotide polymorphism (SNP) rs16944 was analyzed between the cases and healthy control (HC) groups. The genotypic distribution of the rs16944 polymorphism, along with its genetic model analysis, is depicted in **Table 3**. When the Cases and HC groups were compared, no significant differences in allele frequencies were observed. The comparison of genotypes between these groups revealed that the OR for the genetic models, codominant (CC vs. CT), dominant (CC vs. CT+TT), and overdominant (CT vs. CC+TT), were significant (OR = 4.6136, 3.4232, and 3.5387, respectively). The highest OR was observed with the codominant model (OR= 4.6136, 95% CI: 1.83-11.66, *p* = 0.0012). Allele and genotypic frequency analyses were also performed to compare the female and male groups within cases and the male groups between cases and HC groups, which showed non-significant differences (NS) (**Supplementary Table 3**). The comparison between cases and HC in females was significant. Concerning the allele frequencies, they did not show a significant difference between the cases versus HC (OR = 1.6471, 95% CI: 0.95–2.84, *p* = 0.0735) (**Supplementary Table 1**). In females, we found a significant difference in the allele frequencies between cases and HC (OR = 1.9093, 95% CI: 1.0–3.8, *p* = 0.0490). Also, the dominant, codominant (CC vs. CT), and overdominant genetic models showed significant differences (*p* = 0.0038, 0.0015, and 0.0044, respectively).

**Table 3:** Genotype distribution of rs16944 polymorphism and the genetic model analysis between cases and controls and female.

rs16944	ALLELES/ GENOTYPES	CASES (n= 57)	HC (n= 50)	OR	95% CI	*p*
HWE		**0.012**	0.39			
ALLELE FREQUENCY	C (C)	72	51			
GENOTYPES	T	42	49	**1.6471**	0.9537-2.8445	NS- 0.0735^a^
	CC	28	11			
	CT	16	29			
	TT	13	10			
GENETIC MODEL						
CODOMINANT 1	CC vs CT (CC)			**4.6136**	1.8261-11.6562	**0.0012^a^**
CODOMINANT 2	CC vs TT					NS^a^
CODOMINANT 3	CT vs TT					NS^a^
DOMINANT	CC vs.	28	11			
	CT + TT (CC)	29	39	**3.4232**	1.4675-7.9851	**0.0044^a^**
RECESSIVE	TT vs.	44	40			
	CC + CT	13	10			NS^a^
OVERDOMINANT	CT vs.	16	29			
	CC + TT	41	21	**3.5387**	1.5810-7.9208	**0.0021^a^**
FEMALE						
		CASES (n= 44)	HC (n= 26)			
ALLELE FREQUENCY	C	53)	23			
(MAF) Minor allele frequency	T	35	29	**1.9093**	1.001-3.822	**0.0490^a^**
GENOTYPES	CC	20	3			
	CT	13	17			
	TT	11	6			
GENETIC MODEL						
CODOMINANT 1	CC vs CT (CC)					**0.0015^b^**
CODOMINANT 2	CC vs TT					NS^b^
CODOMINANT 3	CT vs TT					NS^a^
DOMINANT	CC vs.	20	3			
	CT + TT (CC)	24	23			**0.0038^b^**
RECESSIVE	TT vs.	33	20			
	CC + CT	11	6			NS^a^
OVERDOMINANT	CT vs.	13	17			
	CC + TT (CCTT	31	9	**4.5043**	1.5992-12.6863	**0.0044**

Allele or genotype associated with IIMs shown in parenthesis.

HC, Healthy controls; HWE, Hardy Weinberg equilibrium; COD, Codominant model; DOM, Dominant model; OD, Overdominant model. NS, non significant. OR; Odds ratio calculation. a) OR (Chi-square). b) Fisher’s exact test. Bold values indicate a significant difference (*p* >0.05).

**Figure 2:**
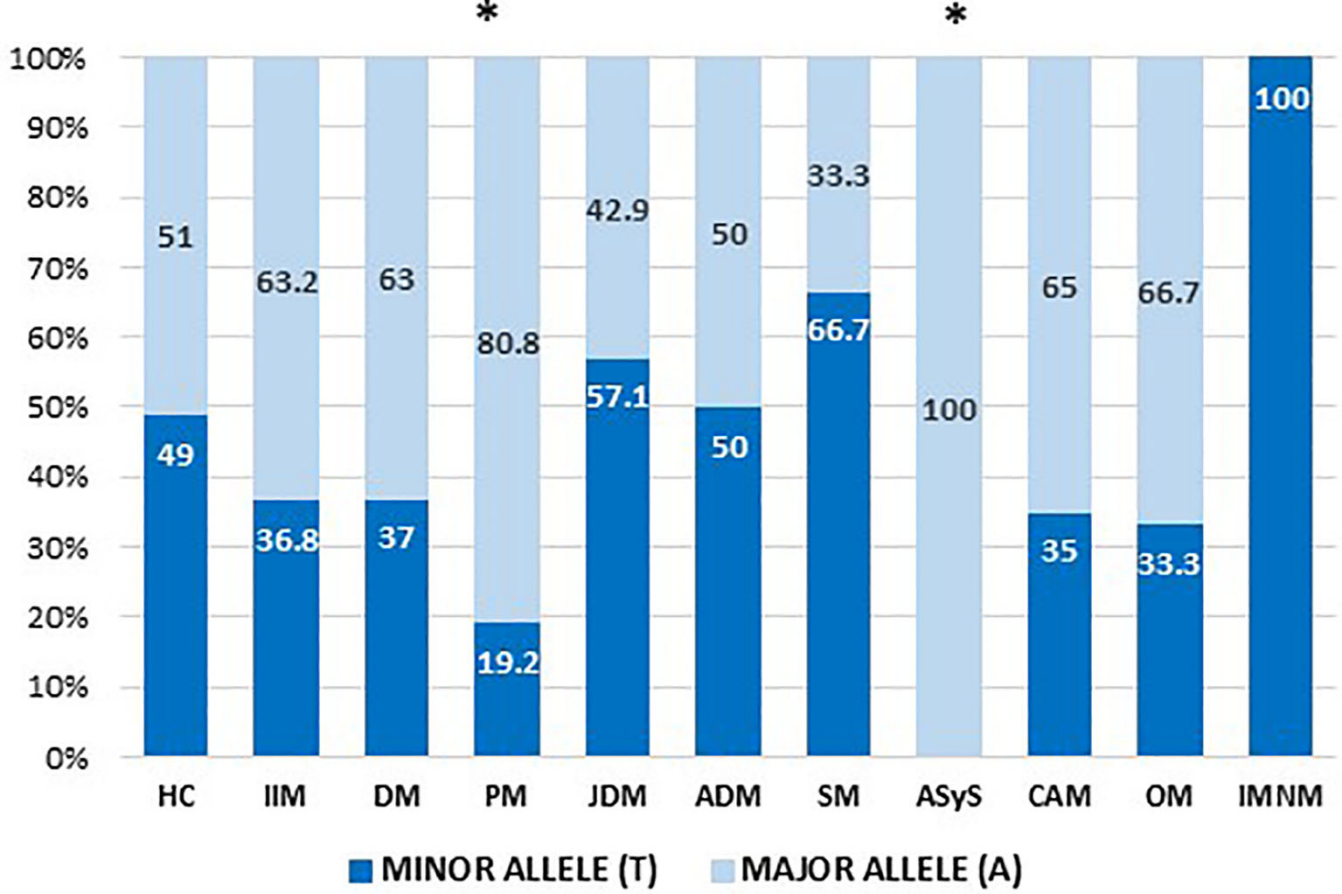
Comparison of the allele frequencies between controls and the different subtypes of IIMs. * >0.05 significance, ** <0.001 significance, *** <0.0001 significance.

There were statistically significant differences among IIMs types; genotypes were compared to those of the HC. DM, PM, ASyS, and CAM showed significant differences. Also, the allele comparison (C) of IIMs against HC alleles showed a difference in the PM patient group (**Figure 2**; **Table 4**).

**Table 4:** Genotype and allele comparison among the types of IIMs against HC.

A.GENOTYPE COMPARISON	OR	CI	*p*
HC vs. DM			
COD (CC vs CT)			**0.0003^a^**
DOM (CC)	**4.4318**	1.611-12.1914	**0.0039^b^**
OD (CC+TT)			**0.0003^a^**
HC vs. PM			
COD (CC vs CT)			**0.0056^a^**
COD (CC vs TT)			**0.0493^a^**
DOM (CC)			**0.0022^a^**
OD (CC+TT)			**0.0319^a^**
HC vs. DM+PM			
COD (CC vs CT)	**9.0390**	3.0356-26.9148	**0.0001^b^**
DOM (CC)	**5.3182**	2.1177-13.3558	**0.0004^b^**
OD (CC+TT)	**6.5102**	2.4184-17.5250	**0.0002^b^**
HC vs. JDM			
DOM (CC)			**0.0106^a^**
OD (CC+TT)			**0.0009^a^**
HC vs. ADM			
OD (CC+TT)			**0.0086^a^**
HC vs. ASyS			
COD (CC vs CT)			**0.0295^a^**
DOM (CC)			**0.0155^a^**
HC vs. CAM			
COD (CC vs CT)			**0.0062^a^**
DOM (CC)			**0.0237^a^**
OD (CC+CT)			**0.0121^a^**

B.ALLELE COMPARISON			
HC vs. DM+PM (C)	**2.1137**	1.1437-3.9065	**0.0169^b^**
HC vs. PM (C)	**4.0353**	1.4104-11.5451	**0.0093^b^**
HC vs. ASyS (C)			**0.0295^a^**

A. Genotype comparisons. B. Allele comparisons. The IIMs not included showed non significant differences (NS).

HC, Healthy controls; DM, Dermatomyositis; PM, Polymyositis; ASyS, Antisynthetase syndrome; CAM, Cancer-associated myositis; COD, Codominant model; DOM, Dominant model; OD, Overdominant model. a) Fisher’s exact test. b) OR (Chi-square). Bold values indicate a significant difference (*p* >0.05).

Regarding the enzyme levels of CPK, AST, ALT, and LDH, no association was found between the enzyme levels and the allele or genotypic frequencies, except for CPK (*p* = 0.0003) (**Table 2**). Yet, a tendency toward lower levels can be observed for CPK and LDH in the TT genotype and for AST and ALT in the CT genotype, but these differences are not significant (NS).

The rs16944 genotypes were compared with the different clinicopathological characteristics in IIM cases (heliotrope rash, V sign, shawl sign, Gottron papules, Holster sign, basal poichioderma, basal panniculitis, basal perionixis, Raynaud phenomenon, basal arthritis, basal hyperpigmentation, periungueal erythema, basal calcinosis, dysphagia, dyspnea, basal arrhythmia, vasculitis, mechanic’s hands, proximal weakness, distal weakness, interstitial lung disease, atrophy, necrosis, abnormal electromyography, inflammation, fat infiltration, endomysial infiltration, perimysial infiltration, and perivascular infiltration). Some of them (Gottron papules, proximal weakness, arthralgia, initial MMT-8 (muscle memory test 8), hypertension, and ALT high levels (>40) were associated with the codominant, dominant, and overdominant genetic models as observed in **Table 5**.

**Table 5:** Genotypes and their association with the clinicopathological characteristics of IIMs patients.

CLINICOPATHOLOGICAL CHARACTERISTICS	GENETIC MODEL	GENOTYPE ASSOCIATED	*p* *
Gottron papules	COD CC vs CT	CC	**0.0498**
Proximal weakness	COD CC vs CT	CC	**0.0368**
	DOM CC vs CT+TT	CC	**0.0461**
	OD CT Vs CC + TT	CC+TT	**0.0390**
Arthralgia	COD	CC	**0.0490**
	OD CT Vs CC + TT t	CC+TT	**0.0382**
Initial MMT-8	COD CC vs CT	CC	**0.0254**
	OD CT Vs CC + TT	CC+TT	**0.0183**
Hypertension	DOM CC vs CT+TT	CC	**0.0339**
ALT >40 FEMALE	COD CC vs CT	CC	**0.0492**
	OD CT Vs CC + TT	CC+TT	**0.0231**

* Fisher’s exact test. MMT-8, Muscle memory test 8. ALT, Alanine aminotransferase. Bold values indicate a significant difference (*p* >0.05).

## Discussion

To our knowledge, this is the first study that associates the *IL1B* rs16944 polymorphism with IIMs. Regarding the autoantibody frequencies obtained in our IIM Mexican patients, the most common were anti-Ro52 (30.4%), anti-Ku (14.3%), anti-SAE1 (12.5%), anti-MDA5 (10.7%), and Anti-TIF1g (10.7%), with the rest displaying frequencies below 10%. These results contrast significantly with those obtained by Gonzalez-Bello et al. in Mexican and Latin American IIM patients in 2021 (25). The most frequent myositis autoantibodies described in that article were Mi-2 (40.1%), Ro52 (25%), Jo-1 (17%), PM75 (10.7%), and Ku (9.8%). These differences could be due to the other IIMs patients from Latin American countries with diverse genetic backgrounds.

About the *IL1B* rs16944 analysis, although the number of cases across different IIMs types (ADM, SM, ASyS, OM, and IMNM) is small, a genotype association is observed in the total IIMs group, which includes DM and PM. We observed that the rs16944 polymorphism is associated with DM, PM, ASyS, and CAM. Particularly, the association with CAM was detected using Fisher’s exact test in the COD model (CC) (*p* = 0.0062), the DOM model (CC) (*p* = 0.0237), and the OD model (CC+CT) (*p* = 0.0121). In this regard, several studies have reported an association between this polymorphism and cancer. Nahar et al. (26), in their meta-analysis publication, reported a significant association with overall cancer in the OD model (OR = 0.86, *p* = 0.044), with breast cancer in the codominant model (CC vs. CT) (OR = 0.86, *p* = 0.043), the codominant model (CT vs. TT) (OR = 1.16, *p* = 0.008) and the OD model (OR = 0.89, *p* = 0.019) (26). Gallegos-Arreola et al. (25) reported an association between the T allele of rs16944 and an increased risk of colorectal cancer (OR = 1.6, *p* = 0.0003) (25). Hoidy et al. (28) reported that the TT genotype of the rs16944 polymorphism is associated with prostate cancer risk (OR = 1.76, 95% CI: 1.18–2.63, *p* = 0.006) (28). Our results with CAM are more congruent with those reported by Hoidy et al. In our study, the C allele had a high frequency in IIMs, although it was not significant (OR = 1.6471, 95% CI: 0.9537–2.8445, *p =* 0.0735). Considering only female cases, when compared with HC, the C allele was significantly associated with cases (OR = 1.9093, 95% CI: 1.001–3822, *p* = 0.0490).

When enzyme levels were analyzed, we observed that the T allele was associated with higher enzyme levels (CPK, AST, ALT, and LDH), indicating that muscle damage and inflammation were more frequent in patients carrying the T allele, whereas the opposite was observed in patients carrying the C allele (Data not shown).

There are several reports of the *IL1B* rs16944 polymorphism in different diseases, such as cancer, keratoconus, type 1 diabetes, and autoimmune diseases like SLE, rheumatoid arthritis, ANCA-associated vasculitis, and antisynthetase syndrome (26–35). Regarding the association between rs16944 and SLE, there are conflicting reports. In 2004, Camargo JF et al. found no significant differences in genotype frequencies of the rs16944 polymorphism between Colombian SLE, RA, and primary Sjögren syndrome (PSS) patients and controls (19). Tahmasebi Z. et al. reported no significant differences in rs16944 genotype frequencies between Iranian SLE patients and controls (31). Behiry EG et al. reported that the TT genotype and the T allele showed significantly higher frequencies in the Egyptian SLE population than in the control group (*p* = 0.005 and < 0.001, respectively). The TT genotype was also significantly associated with several clinical characteristics in SLE (32). On the other hand, Rzeszotarska E et al. (21) reported that the CT genotype in the codominant model, the CT+TT genotype in the dominant model, and the CT genotype in the overdominant model occur more frequently in SLE patients (*p* = 0.0002, 0.0003, and 0.0004, respectively).

Regarding the role of the *IL1B* rs16944 polymorphism in rheumatoid arthritis (RA), a 2020 study found that this polymorphism was associated with an increased risk of RA in both the codominant and recessive models (*p* = 0.029 and 0.021) (33). Harrison P et al. reported in 2008 a statistically significant association between the CC genotype and the C allele with RA in a meta-analysis of pooled data (38). In 2012, a study conducted with a Mexican population of individuals with chronic periodontitis (CP) and rheumatoid arthritis (RA) revealed that the CC genotype was associated with the CP+RA group (39). Additionally, a 2020 study in the Mexican population examining the role of rs16944 in ASyS patients found no significant differences between ASyS patients and the control group (35). Regarding the results of this study, after comparing genotypes between the IIMs and healthy control groups, the CC and CT genotypes differed significantly (*p* = 0.0064 and 0.0034, respectively). Additionally, the OR for the genetic models, dominant, codominant (CC vs. CT and overdominant were significant; OR = 2.482, 4.187, and 3.249, respectively). The highest OR was observed with the codominant model (OR = 4.187, 95% CI: 1.67–10.53, *p* = 0.0023). Our results are consistent with those reported by Rzeszotarska et al. (21) for SLE patients from Poland. Results from various studies in diverse populations suggest that the CC and CT genotypes are most commonly associated with autoimmune diseases, although conflicting findings have been reported. Separately, in 2012, Sandoval-Garcia et al. described an association between the X allele of the *ACTN3* R577X polymorphism and IIMs, particularly with DM, marking the first gene polymorphism identified as associated with IIMs in Mexicans (36).

In this work, we observed that the rs16944 polymorphism is associated with IIMs and, individually, with DM, PM, ASyS, and CAM. Particularly, the association with CAM was detected using Fisher’s exact test in the COD model (CC) (*P* = 0.0062), the DOM model (CC) (*P* = 0.0237), and the OD model (CC+CT) (*P* = 0.0121). In this regard, several studies have reported an association between this polymorphism and cancer. Nahar et al., in their meta-analysis publication, reported a significant association with overall cancer in the OD model (OR = 0.86, *p* = 0.044), with breast cancer in the codominant model (CC vs. CT) (OR = 0.86, *p* = 0.043), the codominant model (CT vs, TT) (OR = 1.16, *p* = 0.008) and the OD model (OR = 0.89, *p* = 0.019) (26). Gallegos-Arreola et al. reported an association between the T allele of rs16944 and an increased risk of colorectal cancer (OR = 1.6, *p* = 0.0003) (25). Hoidy et al. reported that the TT genotype of the rs16944 polymorphism is associated with prostate cancer risk (OR = 1.76, 95% CI: 1.18–2.63, *p* = 0.006) (28). Our results with CAM are more similar to those reported by Hoidy et al.

Similarly, there are reports of an association between the T allele of the rs16944 polymorphism and systemic sclerosis (SS) (OR = 2.538, CI: 1.245–5.177, *p* = 0.011) (37). However, in this study, we did not find any allelic or genotypic association with SM, a condition that shares features with SS, such as vascular damage and fibrosis.

In this study, we conducted an association analysis of the *IL1B* rs16944 CC (codominant and dominant models) and CCTT (overdominant) genotypes with IIMs, enzyme levels, and clinicopathological features in our IIMs patients, constituting the first report of this polymorphism’s association with IIMs.

## Data Availability

The datasets presented in this study can be found in online repositories. The names of the repository/repositories and accession number(s) can be found in the article/**Supplementary Material**.
