# Supplementary material for: Association between IL1B rs16944 polymorphism and the risk of idiopathic inflammatory myopathies
Source: Front Immunol. 2026 Apr 1;17:1697044. doi: 10.3389/fimmu.2026.1697044 (PMC13079294; doi:10.3389/fimmu.2026.1697044)
Supplement: Supplementary file 1 [file Table1.docx]

**Table 1. Antinuclear antibodies (ANAs) in IIMs patients.**

| **ANTINUCLEAR ANTIBODIES (ANAs)** | **TOTAL**  **n (%)** | **GENDER** | | ***p*** | **GENOTYPES** | | | **MODEL** | ***p*** |
| --- | --- | --- | --- | --- | --- | --- | --- | --- | --- |
|  |  | **FEMALE n (%)** | **MALE n (%)** |  | **CC n (%)** | **CT n (%)** | **TT n (%)** |  |  |
| **AC-1 Nuclear homogeneous** | 5 (7.1) | 3 (6.3) | 2 (13.3) | NS | 4 (14.3) | 1 (6.3) | 0 (0) | ALL | NS |
| **AC-3 Centromere** | 1 (1.4) | 1 (2.1) | 0 (0) | NS | 0 (0) | 0 (0) | 1 (7.7) | ALL | NS |
| **AC-4 Nuclear fine speckled** | 28 (40) | 24 (50) | 4 (26.7) | **0.0047^a^** | 16 (57.1) | 5 (31.3) | 7 (53.8) | COD 1  CC vs. CT | **0.0155^b^** |
| **AC-8 Homogeneous nucleolar** | 3 (4.3) | 1 (2.1) | 2 (13.3) | NS | 1 (3.6) | 1 (6.3) | 1 (7.7) | ALL | NS |
| **AC-19 Cytoplasmic dense fine speckled** | 6 (8.6) | 4 (8.3) | 2 (13.3) | NS | 4 (14.3) | 2 (12.5) | 1 (7.7) | ALL | NS |
| **AC-21 Cytoplasmic reticular/ AMA** | 7 (10) | 5 (10.4) | 2 (13.3) | NS | 5 (17.9) | 2 (12.5) | 1 (7.7) | ALL | NS |
| **AC-26 NuMA-like** | 1 (1.4) | 1 (2.1) | 0 (0) | NS0 | 1 (3.6) | 0 (0) | 0 (0) | ALL | NS |
| **AC-28 Mitotic chromosomal** | 1 (1.4) | 0 (0) | 1 (6.7) | NS | 0 (0) | 0 (0) | 0 (0) | ALL | NS |
| **AC-0 Negative** | 11 (15.7) | 9 (18.8) | 2 (13.3) | NS | 3 (10.7) | 5 (31.3) | 3 (23.1) | ALL | NS |
| **Mixed** | 7 (10) | 6 (12.5) | 1 (6.7) | NS | 7 (25) | 0 (0) | 1 (7.7) | COD 1  CC vs. CT | **0.0298^a^** |
| **Positive** | 52 (84.3) | 39 (79.2) | 13 (86.7) | NS | 31 (91.2) | 11 (68.8) | 11 (78.6) | COD 1  CC vs. CT | **0.0459** |
| **TOTAL** | 70 | 54 (77.1) | 16 (22.9) | **<0.0001^a^** | 34 (53.1) | 16 (25) | 14 (21.9) |  |  |

AMA, Anti-mitochondrial antibodies; NuMA, Nuclear mitotic apparatus. COD, Codominant Model. a) Chi-square test. b) Fisher’s exact test. Bold values indicate a significant difference (*p* >0.05).

**Table 2. Myositis specific and associated autoantibodies in IIM patients.**

| **MYOSITIS AUTOANTIBODIES** | **IMM PATIENTS n= 56** | **FEMALE** | **MALE** | ***p*** | **CC**  **(n= 28)** | **CT**  **(n= 16)** | **TT**  **(n= 12)** | ***p*** | **CC+CT** | **TT** | ***p*** |
| --- | --- | --- | --- | --- | --- | --- | --- | --- | --- | --- | --- |
|  |  | **n= 43** | **n= 13** |  | **n** | **n** | **n** |  |  |  |  |
| **All MSAs** | **42 (59.2)**  **(n, %)** | 32 | 9 | NS | 21 | 9. | 12 | NS | 30 | 12 | **0.0256** |
| **Anti-SAE1** | 7 (12.5) | 6 | 1 | NS | 2 | 3 | 2 | NS | 5 | 2 | NS |
| **Anti-MDA5** | 6 (10.7) | 4 | 2 | NS | 2 | 2 | 2 | NS | 4 | 2 | NS |
| **Anti-TIF1g** | 6 (10.7) | 4 | 1 | NS | 3 | 1 | 2 | NS | 4 | 2 | NS |
| **Anti-Mi-2ª** | 5 (8.9) | 3 | 2 | NS | 3 | 1 | 1 | NS | 4 | 1 | NS |
| **Anti-Mi-2b** | 5 (8.9) | 4 | 1 | NS | 4 | 1 | 0 | NS | 5 | 0 | NS |
| **Anti-Jo1** | 4 (7.1) | 2 | 2 | NS | 4 | 0 | 0 | NS | 4 | 0 | NS |
| **Anti-PL-7** | 3 (5.4) | 3 | 0 | NS | 2 | 0 | 1 | NS | 2 | 1 | NS |
| **Anti-EJ** | 2 (3.6) | 2 |  | NS | 1 | 0 | 1 | NS | 1 | 1 | NS |
| **Anti-OJ** | 2 (3.6) | 2 |  | NS | 0 | 0 | 2 | NS | 0 | 2 | **0.0428** |
| **Anti-NXP2** | 1 (1.8) | 1 |  | NS | 0 | 1 | 0 | NS | 1 | 0 | NS |
| **Anti-SRP** | 1 (1.8) | 1 |  | NS | 0 | 0 | 1 | NS | 0 | 1 | NS |
| **Anti-PL-12** | 0 (0) | 0 |  | NS | 0 | 0) | 0 | NS | 0 | 0 | NS |
| **All MAAs** | **29 (40.8)** | **25** | **3** | NS | 11 | 9 | 8 | NS | 20 | 8 | NS |
| **Anti-Ro52** | 17 (30.4) | 14 | 2 | NS | 6 | 6 | 4 | NS | 12 | 4 | NS |
| **Anti-Ku** | 8 (14.3) | 8 | 0 | NS | 5 | 1 | 2 | NS | 6 | 2 | NS |
| **Anti-PM100** | 3 (5.4) | 2 | 1 | NS | 0 | 1 | 2 | NS | 1 | 2 | NS |
| **Anti-PM75** | 1 (1.8) | 1 | 0 | NS | 0 | 1 | 0 | NS | 1 | 0 | NS |
| **POSITIVE** | 36 (50.7) | 30 | 6 | NS | 17 | 10 ( | 9 | NS | 27 | 9 | NS |
| **NEGATIVE** | 20 (28.2) | 13 | 7 | NS | 11 | 6 | 3 | NS | 17 | 3 | NS |
| **MIXED** | 19 (26.8) | 17 | 2 | NS | 13 | 4 | 6 | NS | 17 | 6 | NS |

Mixed: more than 1 autoantibody**.** Analysis performed with Fisher’s exact test.

**Table 3.** **Genotype distribution of rs16944 polymorphism and the genetic model analysis in patients and male.**

| **rs16944** | **ALLELES & GENOTYPES** | **PATIENTS**  **(n= 57)** | | **OR** | **95% CI** | ***p*** |
| --- | --- | --- | --- | --- | --- | --- |
|  |  | **FEMALE**  **(n= 44)** | **MALE**  **(n= 13)** |  |  |  |
| **ALLELE FREQUENCY** | **C** | 54 | 18 |  |  |  |
| **(MAF) minor allele frequency** | **T** | 34 | 8 | 1.4167 | 0.5551-3.6154 | NS^a^ |
| **GENOTYPE (n, %)** | **CC** | 21 | 7 |  |  | NS^b^ |
|  | **CT** | 12 | 4 |  |  | NS^b^ |
|  | **TT** | 11 | 2 |  |  | NS^b^ |
|  |  |  |  |  |  |  |
|  |  |  |  |  |  |  |
| **GENETIC MODEL**  **CODOMINANT 1** | **CC VS. CT** | 20, 13 | 7, 4 | 1.0000 | 0.2140-3.6119 | NS^a^ |
| **CODOMINANT 2** | **CC VS. TT** | 20, 11 | 7,2 | 1.8333 | 0.3242-10.3677 | NS^a^ |
| **CODOMINANT 3** | **CT VS. TT** | 13, 11 | 4,2 | 1.6923 | 0.2588-11.0656 | NS^a^ |
| **DOMINANT** | **CC VS CT + TT** | 21, 23 | 7, 6 | 1.2778 | 0.3696-4.4180 | NS^a^ |
| **RECESSIVE** | **CC + CT VS TT** | 33, 11 | 11, 2 | 1.8333 | 0.3507-9.5843 | NS^a^ |
| **OVERDOMINANT** | **CT VS. CC + TT** | 12, 32 | 4, 9 | 1.1852 | 0.3067-4.5801 | NS^a^ |
| **MALE** | | | | | | |
|  |  | **PATIENTS (n= 13)** | **HEALTHY CONTROLS**  **(n= 24)** |  |  |  |
| **GENOTYPE (n, %)** | **CC** | 7 | 8 |  |  | NS^b^ |
|  | **CT** | 4 | 12 |  |  | NS^b^ |
|  | **TT** | 2 | 4 |  |  | NS^b^ |
| **ALLELE FREQUENCY** | **C** | 18 | 28 |  |  | NS^b^ |
|  | **T** | 8 | 20 | 1.6071 | 0.5845-4.4187 | NS^b^ |
| **GENETIC MODEL**  **DOMINANT** | **CC VS CT + TT** | 7, 6 | 8, 16 | 2.3333 | 0.5860-9.2915 | NS^b^ |
| **RECESSIVE** | **CC + CT VS TT** | 11, 2 | 20, 4 | 1.1000 | 0.1730-6.9958 | NS^b^ |
| **CODOMINANT 1** | **CC VS. CT** | 7, 4 | 8, 12 | 2.625 | 0.5743-11.9987 | NS^b^ |
| **CODOMINANT 2** | **CC VS. TT** | 7, 2 | 8, 4 | 1.7500 | 0.2422-12.6422 | NS^b^ |
| **CODOMINANT 3** | **CT VS. TT** | 4, 2 | 12, 4 | 1.5000 | 0.1950-11.5363 | NS^b^ |
| **OVERDOMINANT** | **CT VS. CC + TT** | 4, 9 | 12, 12 | 2.2500 | 0.5417-9.3450 | NS^b^ |

1. Chi-square. b) Fisher’s exact test.
