# Supplementary figures and images for: Association between IL1B rs16944 polymorphism and the risk of idiopathic inflammatory myopathies
Source: Front Immunol. 2026 Apr 1;17:1697044. doi: 10.3389/fimmu.2026.1697044 (PMC13079294; doi:10.3389/fimmu.2026.1697044)

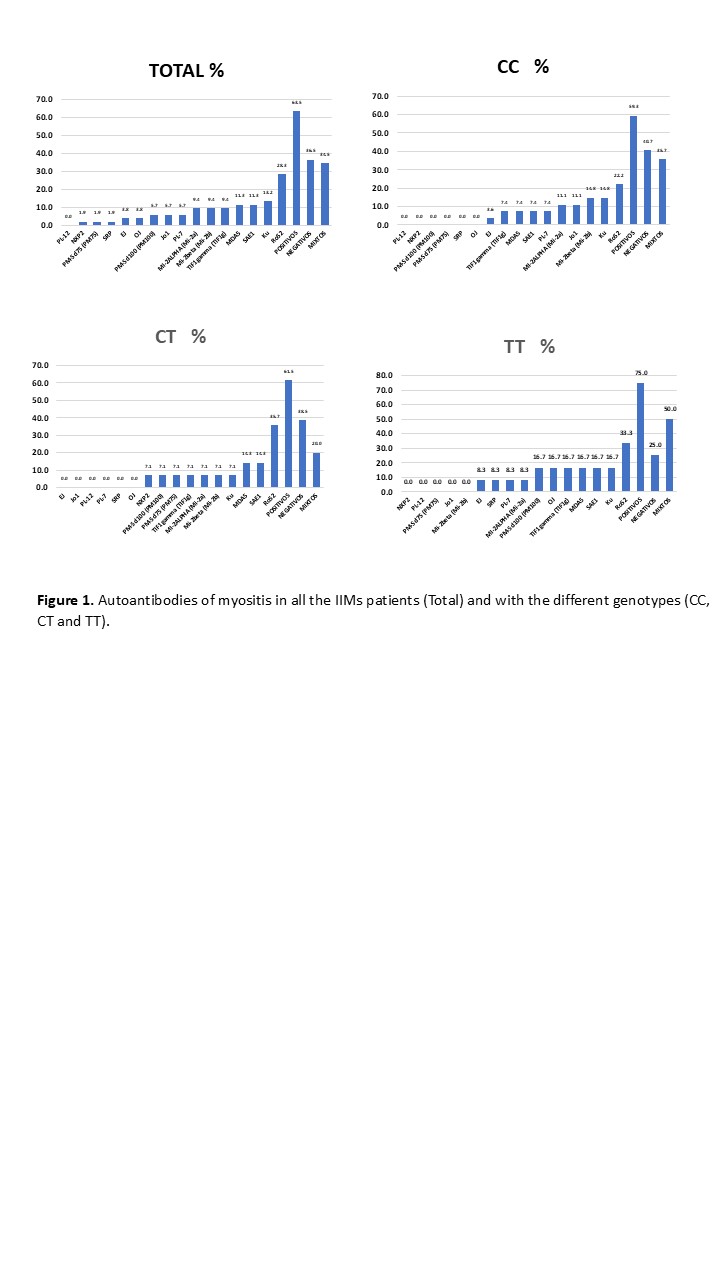

Supplement: Supplementary file 2 [file Image1.jpg]
